# Hegeman & Co.

**Published:** 1868-11-15

**Authors:** 


***Hegeman & Co.***

Will accept our thanks for exquisite specimens of Cod Liver Oil and the Elixirs of Calisaya and Ferrated Calisaya. It would seem impossible to surpass them in perfection of preparation.

